# The Effect of Coronavirus Disease-19 Pandemic Lockdown and the Overlapping Ramadan Fasting Period on Glucose Control in Insulin-Treated Patients With Diabetes: A Flash Glucose Monitoring Study

**DOI:** 10.3389/fnut.2022.843938

**Published:** 2022-03-31

**Authors:** Radwa Helal, Tanveer Ashraf, Maria Majeed, Nader Lessan

**Affiliations:** Imperial College London Diabetes Centre, Abu Dhabi, United Arab Emirates

**Keywords:** Ramadan fasting, type 1 diabetes, type 2 diabetes, flash glucose monitoring, COVID-19, lockdown

## Abstract

**Background:**

A strict lockdown was enforced during coronavirus disease (COVID-19) pandemic in many countries including the UAE. Lockdown period overlapped with Ramadan which is accompanied by its own drastic changes in lifestyle that include meal timings.

**Aims:**

We report the impact of COVID-19 lockdown (between 22/3/2020 and 24/6/2020) on glucose control pre- and postlockdown and during Ramadan, in patients with type 1 diabetes (T1D) and type 2 diabetes (T2D) on insulin therapy.

**Methods:**

A number of twenty-four patients (19 men, 6 women) who were monitoring their glucose levels using flash glucose monitoring (FGM) and remotely connected to the diabetes clinic in Imperial College London Diabetes Centre (ICLDC), Abu Dhabi, UAE were included. Using the international consensus on the use of continuous glucose monitoring guidelines, analyses of data were performed on glucose management indicator (GMI), time in range (TIR), time in hyperglycemia, time in hypoglycemia, low blood glucose index (LBGI) and high blood glucose index (HBGI). Variables were calculated for each period: 30 days before lockdown 14/2/2020–14/3/2020, 30 days into lockdown and pre-Ramadan 20/3/2020–18/4/2020, and 30 days into lockdown and Ramadan 24/4/2020–23/5/2020, using cgmanalysis package in R-studio software.

**Results:**

Mean average glucose (MAG) remained steady before and during lockdown, and no significant differences were observed in TIR, time in hypoglycemia, and LBGI between prelockdown and lockdown periods. However, there was a statistically significant difference in GMI and percentage of time in hyperglycemia (>10.0 mmol/L) between Ramadan and pre-Ramadan during the lockdown period in *p* = 0.007, 0.006, and 0.004, respectively. Percentage of TIR (3.9–10.0 mmol/L) was significantly lower in Ramadan as compared to pre-Ramadan (50.3% vs. 56.1%; *p* = 0.026). Mean absolute glucose (MAG) (182.0 mmol/L vs. 166.6 mmol/L, *p* = 0.007) and HBGI (10.2 (6.8, 14.8) vs. 11.9 (7.9, 17.8), *p* = 0.037) were significantly higher in Ramadan compared to pre-Ramadan period. There was no statistically significant difference in percentage of time in hypoglycemia (<3.9 mmol/L) and LBGI between Ramadan and pre-Ramadan periods.

**Conclusion:**

The lockdown period had no significant effects in the markers of glycemic control in the population studied. However, Ramadan fasting period embedded within this time was associated with several changes that include increase in GMI, HBGI, and glycemic variability similar to what has been reported in other Ramadan studies.

## Introduction

Fasting during the lunar month of Ramadan is a religious responsibility for adult Muslims which entails daytime fasting for 29–30 days. Ramadan fasting is one of the five pillars of Islam. Several groups are exempted from this obligation (including acute and/or chronic illnesses). However, people included in these exemptions, which include some patients with diabetes often choose to proceed with Ramadan fasting for personal, social and cultural reasons, and their individual perceptions of religious law (1). People with diabetes are generally confronted with serious risks such as hypoglycemia and hyperglycemia. The Epidemiology of Diabetes and Ramadan (EPIDIAR) survey of people with diabetes in 13 Islamic countries revealed that around 43% of people with type 1 diabetes mellitus (T1D) and 79% of people with type 2 diabetes mellitus (T2D) fast during Ramadan despite the presence of exemptions (2).

Ramadan in the year 2020 was very different from previous years due to the outbreak of coronavirus disease (COVID-19). The COVID-19 pandemic response in many countries included several restrictions. In the United Arab Emirates (UAE), a strict lockdown was enforced between 22 March 2020 and 24 June 2020. The lockdown period overlapped with Ramadan fasting which lasted for 30 days in UAE and with around 14 h of fasting per day. Ramadan began on 23 April 2020 and ended on the 23 May 2020.

Ramadan is inevitably associated with its own drastic changes in lifestyle that include sudden change in mealtimes, sleep, and routine daily activity. The first meal in Ramadan day (*Iftar*) consumed after a period of fasting is usually of high-calorie diet which might contribute to hyperglycemia (3). In normal individuals, there is a slight drop in average blood glucose levels in the beginning of Ramadan followed by stabilization through the second half of the month. Yet, all variations are within physiological range (4).

Patients with diabetes who tend to continue their routine medications usually build up episodes of hypoglycemia depending upon medication compliance, type of consumed food, modifications in physical activities, or binge eating after the *Iftar* meal (5, 6). Some previous studies showed conflicting changes in the overall glycemic control during Ramadan as compared to pre-Ramadan period (3, 7–10).

Our previous study (11) reported no significant differences in markers of overall glycemic control and in number of high or low glucose excursions between pre-Ramadan and Ramadan periods. Moreover, the absolute differences in CGM parameters during the pre-Ramadan and Ramadan periods were very small. Cultural, personal, social, nutritional, and medical factors may contribute to this variation. This emphasizes the implication of tailored individualized plan for patients which focuses on meal types and timing, and also making appropriate medication and dose changes during Ramadan fasting period.

Although some studies noted a decrease in food consumption and healthier diet practices during the lockdown period (12–15), many studies found either an increase in snacking and meal numbers or an increase in unfavorable food choices and dietary habits (16–19). Therefore, COVID-19 lockdown resulted in both favorable and unfavorable changes in eating practices, and this may have both short- and long-term consequences on health. The positive diet practices included an increase in the consumption of fresh produce, mostly fruits and vegetables, and an increase in home cooking during lockdown. However, poor food habits were seen in most studies, which include increased snacking and meal frequency, reduced fresh production, and increased comfort foods. Reasons for changes in behavior predominately included limited availability and increased price. This was associated with mental health conditions that include depression and anxiety, sedentary time, and weight gain (20). For patients with diabetes, stable rhythm of lifestyle proposes improvement in glucose control with less need to dosing changes in antidiabetic treatment (21).

Recently, some studies presented the effect of COVID-19 lockdown in glycemic control in patients with diabetes using CGM; nevertheless, majority of these studies done in countries do not practice Ramadan fasting, which make that their results on lockdown impact are independent from Ramadan fasting influence on glycemic control (22, 23). The fact that diabetes has been reported in the media as a risk factor for COVID-19 prognosis may have contributed to the improvement in glycemic management into lockdown.

The purpose of this study is to assess to what extent the lockdown overlapped with Ramadan affected ambulatory glucose metrics measured by FGM devices, as defined by the international consensus recommendation guidelines on clinical targets for continuous glucose monitoring data interpretation (24). To assess this, we compared the glycemic profile of patients with type 1 and insulin-treated type 2 diabetes using flash glucose monitoring (FGM), before and during lockdown including the holy month of Ramadan. In addition, we explored the clinical and demographic factors associated with a decline in glycemic control across this period through retrospective access of electronic medical records (EMRs) on Imperial College London Diabetes Centre’s database (ICLDC). Results and conclusion from this study could be used in future for better diabetes management in Ramadan during the COVID-19 restrictions, which considers the sociocultural issues relevant to eventual circumstances.

## Participants and Methods

### Study Design and Participants

This is a retrospective observational single-centre study based on data retrieved from EMRs for patients using FGM (FreeStyle Libre, Abbott, Witney, United Kingdom) who have linked their glucose data to ICLDC using the LibreView online platform.^1^ Of the patients with diabetes who are LibreView users, we identified a cohort of 50 patients with glucose profile data uploaded within the period of 12/2/2020–23/5/2020 to include at least 30 days before COVID-19 lockdown, 30 days into lockdown and pre-Ramadan, and 30 days into lockdown and Ramadan. Of note, during the period of lockdown, regular lifestyle was maintained since COVID-19 infection spread was very limited in the UAE.

### Outcome Measures

The main study outcomes were the assessment of FGM glycemic variables and to compare them in between the 3 time periods (before lockdown, into lockdown and pre-Ramadan, and into lockdown and Ramadan). These variables include mean average glucose (MAG), glucose management indicator (GMI), estimated A1c, interquartile ranges of glucose, coefficient of variation (CV), time spent in range (TIR) (70-180 mg/dL, 3.9-10.0 mmol/L), time spent in hypoglycemia level 1 (54–70 mg/dL, 3.9–3.0 mmol/L), time spent in hypoglycemia level 2 (<54 mg/dL, <3.0 mmol/L), time spent in hyperglycemia level 1 (180–250 mg/dL, 10.0–13.9 mmol/L), time spent in hyperglycemia level 2 (>250 mg/dL, >13.9 mmol/L), mean amplitude of glycemic excursions (MAGE), mean of daily differences (MODD), low blood glucose index (LBGI), high blood glucose index (HBGI), and area under the curve (AUC). These variables were calculated using cgmanalysis package in R-studio software (25). In addition to this, we compared change across a range of glycemic variables between February and May 2020 for each of the following periods: 30 days before COVID-19 lockdown 4/2/2020–14/3/2020 (period 1), 30 days into lockdown and pre-Ramadan 20/3/2020–18/4/2020 (period 2), and 30 days into lockdown and Ramadan 24/4/2020–23/5/2020 (period 3).

### Statistical Analysis

A number of three time periods were compared using repeated measures of ANOVA, with Greenhouse-Geisser correction for significance level (*p* = 0.05) using STATA version 15.0. All pairwise comparisons were adjusted with Bonferroni method in *post hoc* analyses. *Post hoc* multiple comparisons were reported for the significant outcomes only, whereas no pairwise comparisons were assessed for measures, where null hypothesis could not be rejected.

## Results

A total of 24 patients with diabetes (19 men and 6 women) had complete data [≥70% FGM sensor data captured (24, 26)] and were included in the study. A total of eighteen had T1D, 5 had insulin-treated T2D, and one patient had maturity onset diabetes of the young (MODY). All patients with T1D were on multiple daily insulin (MDI) regimen, with mean total daily insulin dose of 90 ± 26 units. Patients with T2D were on insulin together with other agents as follows: metformin (*n* = 1), gliptin with metformin (*n* = 3), or dapagliflozin (*n* = 1). The mean total daily insulin dose in this group was 56 ± 24 units. The patient with MODY was on MDI regimen with a daily insulin dose of 46 units.

Data were categorized and analyzed primarily to compare FGM metrics before COVID-19 lockdown 1/1/2020–11/3/2020 and during COVID-19 lockdown 12/3/2020–15/5/2020 (which included pre-Ramadan and Ramadan month) within 60 days for each period (Table 1). MAG remained steady before and during lockdown, with no significant differences observed in TIR, time in hypoglycemia, and LBGI between prelockdown and lockdown periods.

**TABLE 1:** Comparison of FGM metrics between *prelockdown and lockdown period.

FGM metrics	Prelockdown	Lockdown	*p*-value
**MAG (mg/dL)	169.4 (45.7)	173.6 (42.9)	0.334
GMI (mmol/L)	7.4 (1.1)	7.5 (1.0)	0.319
Estimated A1c	7.5 (1.6)	7.7 (1.5)	0.358
CV	0.38 (0.1)	0.39 (0.1)	0.184
Percentage of TIR	54.3 (20.9)	53.0 (18.6)	0.508
Percentage of TBR level1, median (IQR)	4.9 (1.8, 10.2)	4.0 (2.5, 8.3)	0.903
Percentage of TBR Level 2	14.5 (6.0, 45.5)	16.0 (9.0, 47.8)	0.269
Percentage of TAR level 1, median (IQR)	39.1 (24.4)	40.7 (22.0)	0.48
MAGE	113.6 (31.5)	118.6 (38.4)	0.291
MODD	18.1 (4.7)	18.1 (4.6)	0.885
LBGI	5.4 (2.8)	5.7 (2.8)	0.473
HBGI, median (IQR)	9.8 (6.5, 15.7)	13.1 (7.2, 16.4)	0.326

*COVID-19 prelockdown period: 1/1/2020–11/3/2020 and lockdown 12/3/2020–15/5/2020 (60 days in each period). Data presented as mean (SD), or median (interquartile range – IQR) as stated.

**MAG, mean average glucose; GMI, glucose management indicator; CV, coefficient of variation; TIR, time in range (defined as 70–180 mg/dL); TBR, time below range (level 1: 54–70 mg/dL; level 2: <54 mg/dL); TAR, time above range (level 1: 180–250 mg/dL; level 2: >250 mg/dL); MAGE, mean amplitude of glycemic excursions; MODD, mean of daily differences; LBGI, low blood glucose index; HBGI, high blood glucose index.

Further analysis of the data was performed based on the comparison of 3 time periods: pre-COVID-19 lockdown, pre-Ramadan (into lockdown), and Ramadan (into lockdown) with 30 days for each period Tables 2, 3.

**TABLE 2:** Comparison of FGM metrics between *pre-Ramadan and Ramadan during lockdown.

FGM Metrics	pre-Ramadan	Ramadan	*p*-value
Estimated A1c	7.4 (1.7)	8.0 (1.5)	0.007
**MAG (mg/dL)	166.6 (47.4)	182.0 (41.9)	0.007
GMI (mmol/L)	7.3 (1.1)	7.7 (1.0)	0.006
CV	0.4 (0.1)	0.4 (0.1)	0.155
TIR	390.8 (136.3)	352.4 (153.1)	0.19
Percentage of TIR	56.1 (19.0)	50.3 (20.4)	0.027
TBR level 1	33.2 (31.8)	22.9 (23.3)	0.07
Percentage of TBR level 1	4.6 (4.5)	3.2 (3.2)	0.05
TBR level 2	25.1 (32.1)	15.7 (22.0)	0.21
Percentage of TBR level 2	3.5 (4.4)	2.2 (3.0)	0.193
TAR level 1	149.6 (76.9)	177.5 (65.1)	0.022
Percentage of TAR level 1	21.3 (10.2)	25.5 (8.4)	0.01
TAR level 2	103.5 (118.3)	132.1 (112.1)	0.097
Percentage of TAR level 2	15.2 (17.5)	19.5 (17.3)	0.045
AUC	115476.0 (33427.3)	125698.4 (30057.3)	0.09
MAGE	115.6 (39.9)	122.8 (36.0)	0.42
MODD	17.8 (5.1)	18.0 (4.2)	1
J_index	57.1 (33.2)	64.8 (29.7)	0.047
LBGI	5.8 (2.8)	5.5 (3.5)	1
HBGI	11.8 (7.8)	13.6 (7.4)	0.037

*During lockdown: pre-Ramadan 20/3/2020–18/4/2020, Ramadan 24/4/2020–23/5/2020 (30 days in each period). Data are presented as mean (SD).

**MAG, mean average glucose; GMI, glucose management indicator; CV, coefficient of variation; TIR, time in range (defined as 70–180 mg/dL); TBR, time below range (level 1: 54–70 mg/dL; level 2: <54 mg/dL); TAR, time above range (level 1: 180–250 mg/dL; level 2: >250 mg/dL); AUC, area under the curve; MAGE, mean amplitude of glycemic excursions; MODD, mean of daily differences; LBGI, low blood glucose index; HBGI, high blood glucose index.

**TABLE 3:** Comparison of FGM metrics during *prelockdown and Ramadan during lockdown.

FGM metrics	Prelockdown	Ramadan (during lockdown)	*p*-value
Estimated A1c	7.5 (1.5)	8.0 (1.5)	0.014
**MAG (mg/dL)	167.8 (43.2)	182.0 (41.9)	0.013
GMI (mmol/L)	7.3 (1.0)	7.7 (1.0)	0.013
CV	0.4 (0.1)	0.4 (0.1)	0.729
TIR	392.7 (153.9)	352.4 (153.1)	0.156
Percentage of TIR	55.1 (20.1)	50.3 (20.4)	0.092
TBR level 1	30.0 (27.1)	22.9 (23.3)	0.225
Percentage of TBR level 1	4.4 (4.0)	3.2 (3.2)	0.133
TBR level 2	20.5 (28.2)	15.7 (22.0)	1
Percentage of TBR level 2	2.9 (3.8)	2.2 (3.0)	0.918
TAR level 1	165.5 (75.9)	177.5 (65.1)	0.701
Percentage of TAR level 1	23.1 (10.1)	25.5 (8.4)	0.281
TAR level 2	107.1 (113.4)	132.1 (112.1)	0.179
Percentage of TAR level 2	15.1 (16.1)	19.5 (17.3)	0.045
AUC	119404.8 (33181.5)	125698.4 (30057.3)	0.525
MAGE	113.8 (34.4)	122.8 (36.0)	0.2
MODD	18.1 (4.7)	18.0 (4.2)	1
J_index	56.4 (28.4)	64.8 (29.7)	0.027
LBGI	5.3 (2.9)	5.5 (3.5)	1
HBGI	11.8 (6.9)	13.6 (7.4)	0.038

*Prelockdown: 14/2/2020–14/3/2020 and Ramadan (during lockdown): 24/4/2020–23/5/2020, 30 days in each period. Data are presented as mean (SD).

**MAG, mean average glucose; GMI, glucose management indicator; CV, coefficient of variation; TIR, time in range (defined as 70–180 mg/dL); TBR, time below range (level 1: 54–70 mg/dL; level 2: <54 mg/dL); TAR, time above range (level 1: 180–250 mg/dL; level 2: >250 mg/dL); MAGE, mean amplitude of glycemic excursions; MODD, mean of daily differences; LBGI, low blood glucose index; HBGI, high blood glucose index; AUC, area under the curve.

Table 2 shows pairwise comparisons during lockdown in pre-Ramadan and Ramadan periods, where estimated a1c was higher in Ramadan as compared to pre-Ramadan, with Bonferroni adjustment (8.0 (1.5) vs. 7.4 (1.7), *p* = 0.007). Moreover, statistically significant difference was observed in MAG, GMI, and time in hypoglycemia level 1, time in hyperglycemia level 1, and j-index.

Percentage of time in range (TIR) was significantly lower as 50.3% in Ramadan as compared to 56.1% pre-Ramadan. Higher MAG was reported in Ramadan (182.0 mmol/L) with significant statistical difference than its level in pre-Ramadan period (166.6 mmol/L). HBGI was also higher during Ramadan whereas no statistically significant differences were observed in percentage of time in hypoglycemia level 2 and LBGI.

In Table 3, which displays pairwise comparisons between pre-COVID-19 lockdown and Ramadan (into lockdown), a similar trend is observed. There is no significant difference intime in range, time in both hypoglycemia and hyperglycemia (levels 1 and 2), AUC, MODD, and LBGI, whereas statistically significant difference was reported for estimated a1c, MAG, GMI, j-index, and HBGI.

Overall comparisons of the three time period studies show that average glucose measure was statistically higher in Ramadan vs. pre-Ramadan and pre-COVID period. Similar trend was reported for GMI, median, 25 and 75th percentiles. Parameters for glucose variability did not show a statistically significant increase in Ramadan as compared to pre-Ramadan or pre-COVID, which include standard deviation (*p* = 0.178) and CV (*p* = 0.145).

There was a significant decline in percentage of TIR in Ramadan as compared to pre-Ramadan only (*p* = 0.027); however, statistical difference was not noted for absolute number of minutes in TIR in both groups (*p* = 0.101). Risk of hyperglycemia using HBGI was significantly higher in Ramadan period (*p* = 0.047) using Friedman’s test for comparison. LBGI, an indicator of risk of hypoglycemia, did not show statistical association in three time periods.

Time below range (TBR) (hypoglycemia levels 1 and 2) was not associated with the changes in three time periods. There is a slight significance in percentage of time above range (TAR) (hypoglycemia level 1). TAR (hyperglycemia level 1) was statistically higher in Ramadan, and the significance was maintained in pairwise comparisons between Ramadan and pre-Ramadan only.

**FIGURE 1:**
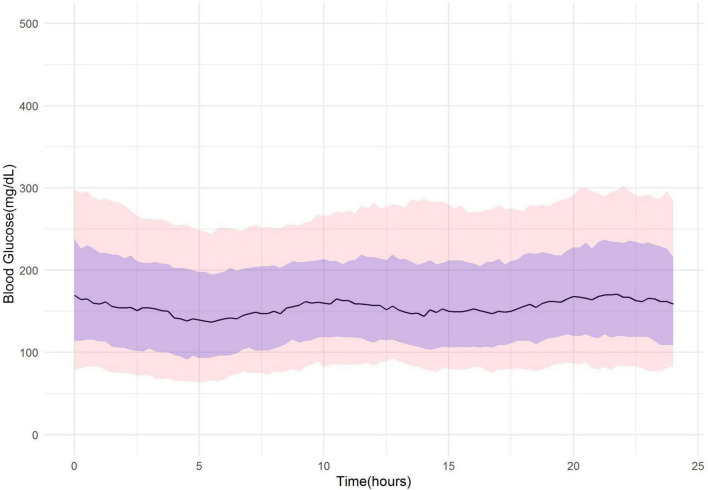
FGM glucose profile (*n* = 24) in 30 days pre-COVID-19 lockdown and pre-Ramadan (14 February 2020–14 March 2020). Line indicates median glucose and purple shaded area shows 25 and 75th percentiles. Pink area denotes 10 and 90th percentiles.

**FIGURE 2:**
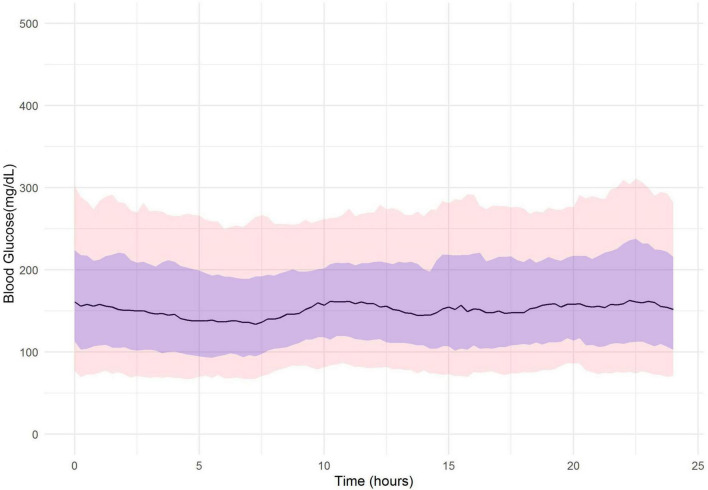
FGM glucose profile (*n* = 24) in 30 days during COVID-19 lockdown and pre-Ramadan (20 March 2020– 18 April 2020). Line indicates median glucose and purple shaded area shows 25 and 75th percentiles. Pink area denotes 10 and 90th percentiles.

**FIGURE 3:**
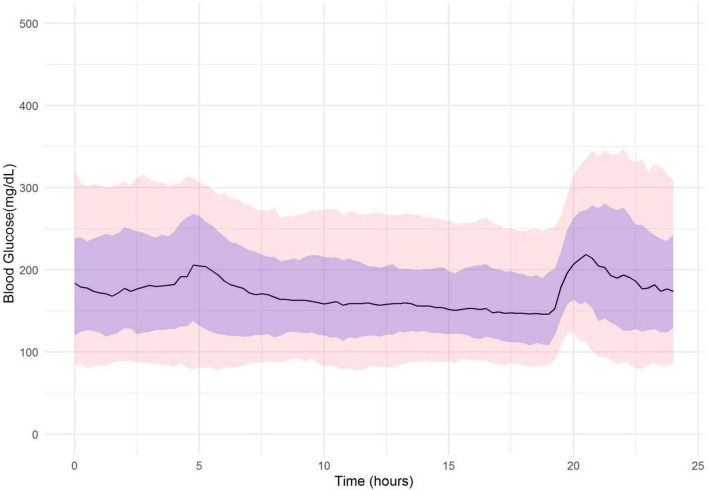
FGM glucose profile (*n* = 24) in 30 days during Ramadan and during COVID-19 lockdown (24 April 2020 – 23 May 2020). Line indicates median glucose and purple shaded area shows 25 and 75th percentiles. Pink area denotes 10 and 90th percentiles.

For further exploratory analysis, we evaluated the glycemic effect of the lockdown by displaying the overall 24-h glucose profile for patients (*n* = 24) in the 3 periods. Similar trend is observed in Figure 1, which shows median glucose level, 10, 25, 75, and 90th percentiles in pre-COVID lockdown, and Figure 2 shows glucose level in pre-Ramadan and lockdown. Figure 3 shows consistent late-night elevation of median glucose level, 10, 25, 75, and 90th percentiles in Ramadan and COVID lockdown period. This indicates the occurrence of hyperglycemia after iftar meal.

**FIGURE 4:**
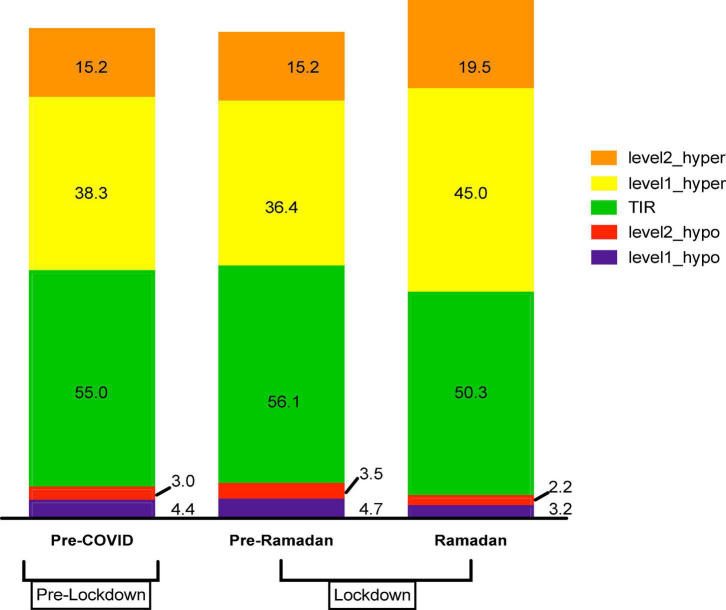
Comparison of percentage of TIRs according to COVID-19 lockdown time points: before lockdown 4/2/2020–14/3/2020 (pre-COVID), during lockdown and pre-Ramadan 20/3/2020–18/4/2020 (pre-Ramadan), and during lockdown and Ramadan 24/4/2020–23/5/2020 (Ramadan). TIR = percentage of TIR (70–180 mg/dL, 3.9–10.0 mmol/L), Level1_hypo = percentage of TBR level 1 (<70–54 mg/dL, <3.9–3.0 mmol/L), Level2_hypo = percentage of TBR level 2 (<54 mg/dL, <3.0 mmol/L), Level1_hyper = percentage of TAR level 1 (>180–250 mg/dL, >10.0 mmol/L), Level2_hyper = percentage of TAR level 2 (>250 mg/dL, >13.9 mmol/L).

Figure 4 shows the comparison of percentage of TIR, TAR (hyperglycemia levels 1 and 2), and TBR (hypoglycemia levels 1 and 2) between before lockdown, pre-Ramadan, and Ramadan after lockdown. Slight decrease occurs in TIR in Ramadan (50.3%) as compared to pre-COVID (55%) and pre-Ramadan (56.1%) (*p* = 0.026). Whereas TAR showed greater increase 45% for hyperglycemia level 1 and 19.5% for hyperglycemia level 2 in Ramadan, with almost the same percentages in pre-COVID and pre-Ramadan, ANOVA test showed statistically significant difference in both levels; hyperglycemia level 1 (*p* = 0.015) 1 and hyperglycemia level 2 (*p* = 0.027).

## Discussion

Coronavirus disease-19 pandemic is an unprecedented healthcare crisis. For the first time in recorded human history, total lockdown and stay-at-home restrictions were imposed and resulted in extreme disruption to lives of people worldwide. The accompanying anxiety was much exaggerated in patients with chronic diseases. This anxiety was further exacerbated in people with diabetes who were shown to be a high-risk group. As a direct unfavorable effect on glycemic control, the COVID-19 lockdown could lead to inactivity and indirect negative effects on glucose control. On the other hand, this lack of activity may be accompanied by changes in food intake and meal patterns with its own effects on glycemia, which may in fact go in either direction.

In this study, we have compared FGM-derived indicators of glycemic control and variability before and during the COVID-19 lockdown period.

The overlap of the Ramadan fasting with the lockdown period was an important consideration in analyzing our data. Therefore, further data analysis has been performed to compare FGM metrics pre-Ramadan and Ramadan, during the lockdown period. Furthermore, a separate analysis was performed to see any differences between prelockdown and Ramadan (into lockdown).

Contrary to our expectation, we found no significant difference in FGM-derived glucose metrics between these two periods. There was no statistical difference in MAGE (mean amplitude of glycemic excursions) between prelockdown, pre-Ramadan, or Ramadan periods in our studies sample. MAGE considers glycemic peaks and nadirs occurring daily without counting the total number of fluctuations (27). Likewise, LBGI did not show statistically significant differences between these three time period studies whereas HBGI was significantly higher in Ramadan than prelockdown and pre-Ramadan periods. Additionally, when we compared Ramadan 2020 and Ramadan 2019, there was no statistically significant differences in FGM-derived indicators of glycemic control and variability.

Ramadan is accompanied by its own sudden and drastic changes in lifestyle that include meal timings. These are accompanied by alterations in sleeping schedules and circadian rhythm of various hormones. For patients with diabetes, there are changes in glycemic variability patterns which are more pronounced in patients on insulin secretagogues and insulin therapy, which includes multiple daily injections (MDIs) of insulin or continuous subcutaneous insulin infusion (CSII). These have been well-described in work by several groups (28), including our own (29). The paradoxical risk of excessive eating after hours of fasting and also reduction in total sleep duration may lead to the increase in glycemic variability (5).

In keeping with the previous work, in this study, we have shown a significant reduction in time spent in range (3.9–10.0 mmol/L), time spent in hypoglycemia and hyperglycemia during Ramadan as compared to pre-Ramadan period, with late-night-time surge of mean blood glucose after *Iftar* time (Figure 3). Moreover, j-index (which represents a measure of the quality of glycemic control based on the combination of information calculated from the mean and SD) was significantly higher in Ramadan than pre-COVID and pre-Ramadan periods. As such, even during the COVID lockdown period, the effects of Ramadan fasting were apparent.

Our results are in concurrence with the study performed in Spain by Beato–Víbora (22), patients with type 1 diabetes using CGM and FGM reported no deterioration in glycemic control related to the prolonged COVID-19 lockdown, and TBR remained unchanged, whereas TIR and estimated HbA1c improved. On the other hand, Bonora et al. (23) and Verma et al. (30) suggested that changes in routine daily activities and having more time for self-management had beneficial effects on glycemic control and consequently diabetes management during lockdown in patients with diabetes, at least in the short term. Another study reported an improvement in glycemic control after 8 weeks of lockdown, especially in patients with reduced baseline control (31).

What about the lockdown effect with Ramadan period dissected out? Our data showed no significant differences in multiple glucose metrics between pre-COVID-19 lockdown and pre-Ramadan periods. This underlines the drastic effect of Ramadan on glucose control and glycemic variability in Muslim patients with diabetes who strictly practice Ramadan fasting. Moreover, in year 2020, time synchronization of COVID-19 lockdown and Ramadan accentuated this influence.

## Conclusion

Our study did not find any relevant significant effect of the lockdown itself on glycemic control in this group of patients. This study also highlights the effects of Ramadan fasting in insulin-treated patients with diabetes, which results in major changes in glycemic profiles, particularly pronounced in the evening hours after the fast is broken. These changes were apparent even within the COVID-19 lockdown period which had its own dramatic lifestyle changes. These findings may have important lessons in designing appropriate lifestyle strategies for management of insulin-treated patients.

## Limitations

This was a single-centre study conducted in specialized diabetes management centre. Moreover, the selection bias may have influenced the glucose excursions, as FGM users in our center tend to have better glycemic control. Therefore, it remains unclear whether these results can be generalized to patients with diabetes with poorer control or not. In addition, only a small sample size of patients was studied, and this might skew the data.

## Data Availability Statement

The original contributions presented in the study are included in the article, further inquiries can be directed to the corresponding author.

## Ethics Statement

The study protocol was approved by Institutional Research Ethical committee in ICLDC (IREC055) and Department of Health (DOH), Abu Dhabi, United Arab Emirates. The study was conducted according to the ethical principles of the Declaration of Helsinki (32).

## Author Contributions

RH: data acquisition, data interpretation, and manuscript writing. TA: data interpretation and manuscript writing. MM: statistical analyses. NL: study design, data interpretation, and manuscript editing. All authors contributed to the article and approved the submitted version.

## Conflict of Interest

The authors declare that the research was conducted in the absence of any commercial or financial relationships that could be construed as a potential conflict of interest.

## Publisher’s Note

All claims expressed in this article are solely those of the authors and do not necessarily represent those of their affiliated organizations, or those of the publisher, the editors and the reviewers. Any product that may be evaluated in this article, or claim that may be made by its manufacturer, is not guaranteed or endorsed by the publisher.

## Footnotes

1
libreview.com
